# Pan-African Genetic Structure in the African Buffalo (*Syncerus caffer*): Investigating Intraspecific Divergence

**DOI:** 10.1371/journal.pone.0056235

**Published:** 2013-02-21

**Authors:** Nathalie Smitz, Cécile Berthouly, Daniel Cornélis, Rasmus Heller, Pim Van Hooft, Philippe Chardonnet, Alexandre Caron, Herbert Prins, Bettine Jansen van Vuuren, Hans De Iongh, Johan Michaux

**Affiliations:** 1 Departement of Life Sciences- Conservation Genetics, University of Liège, Liège, Belgium; 2 Centre de Coopération Internationale en Recherche Agronomique pour le Développement (CIRAD), Campus International de Baillarguet, Montferrier-le-Lez, France; 3 Department of Biology- Bioinformatics, University of Copenhagen, Copenhagen, Denmark; 4 Resource Ecology Group, Wageningen University, Wageningen, The Netherlands; 5 International Foundation for the Conservation of Wildlife (IGF), Paris, France; 6 Department Environment and Societies- Centre de Coopération Internationale en Recherche Agronomique pour le Développement (CIRAD), University of Zimbabwe, Harare, Zimbabwe; 7 Department of Zoology and Entomology- Mammal Research Institute, University of Pretoria, Pretoria, South Africa; 8 Tropical Nature Conservation and Vertebrate Ecology Group, Wageningen University, Wageningen, The Netherlands; 9 Department of Zoology- Centre for Invasion Biology, University of Johannesburg, Johannesburg, South Africa; 10 Institute of Environmental Sciences, Leiden University, Leiden, The Netherlands; 11 Centre de Biologie et de Gestion des Populations (CBGP), Campus International de Baillarguet, Montferrier-le-Lez, France; University of York, United Kingdom

## Abstract

The African buffalo (*Syncerus caffer*) exhibits extreme morphological variability, which has led to controversies about the validity and taxonomic status of the various recognized subspecies. The present study aims to clarify these by inferring the pan-African spatial distribution of genetic diversity, using a comprehensive set of mitochondrial *D-loop* sequences from across the entire range of the species. All analyses converged on the existence of two distinct lineages, corresponding to a group encompassing West and Central African populations and a group encompassing East and Southern African populations. The former is currently assigned to two to three subspecies (*S. c. nanus*, *S. c. brachyceros*, *S. c. aequinoctialis*) and the latter to a separate subspecies (*S. c. caffer*). Forty-two per cent of the total amount of genetic diversity is explained by the between-lineage component, with one to seventeen female migrants per generation inferred as consistent with the isolation-with-migration model. The two lineages diverged between 145 000 to 449 000 years ago, with strong indications for a population expansion in both lineages, as revealed by coalescent-based analyses, summary statistics and a star-like topology of the haplotype network for the *S. c. caffer* lineage. A Bayesian analysis identified the most probable historical migration routes, with the Cape buffalo undertaking successive colonization events from Eastern toward Southern Africa. Furthermore, our analyses indicate that, in the West-Central African lineage, the forest ecophenotype may be a derived form of the savanna ecophenotype and not vice versa, as has previously been proposed. The African buffalo most likely expanded and diverged in the late to middle Pleistocene from an ancestral population located around the current-day Central African Republic, adapting morphologically to colonize new habitats, hence developing the variety of ecophenotypes observed today.

## Introduction

The phylogeographic pattern of most of the savanna mammals distributed across Africa can be partitioned into two to four main lineages. These lineages are typically associated with a West-Central, Eastern, Southern and/or South-East African distribution. For example, the hartebeest (*Alcelaphus buselaphus*) and the common warthog (*Phacochoerus africanus*) comprise three lineages associated with West-Central, Eastern and Southern Africa [1], [2]. The wildebeest (*Connochaetes taurinus*), the topi (*Damaliscus lunatus*), the greater kudu (*Tragelaphus strepsiceros*), and the wild dog (*Lycaon pictus*) comprise at least two lineages associated with Eastern and Southern Africa [1], [3]–[5]. A partitioning into two lineages between West-Central and East-Southern Africa is observed for the kob (*Kobus kob*), the African lion (*Panther leo*), the roan antelope (*Hippotragus equinus*), the waterbuck (*Kobus ellipsiprymnus*), the savanna elephant (*Loxodonta africana*) and the bushbuck (*Tragelaphus scriptus*) [6]–[12]. Species that have a largely East to Southern (including South-West) African distribution often divide into two or three lineages, such as impala (*Aepyceros melampus*) which has a distinct South-West African lineage [13], or sable antelope (*Hippotragus niger*) which has two East African lineages, a South-West lineage and a Southern lineage [14]–[16]. The congruency of phylogeographic patterns among taxonomic groups and trophic levels is generally attributed to the existence of similar forces shaping the evolutionary history of species, with common African refugia [1], [7], [9], [17] and speciation events being climatically mediated [18]. Paleoclimatic reconstructions show that the African continent was marked by pronounced monsoonal changes during the Pleistocene (*i.e.,* climatic oscillations), paced by earth’s orbital variations [18]. The vegetation changes associated with these climatic changes were probably the main driver of population expansions in savanna species during cool and dry phases, and population contraction during wet and warm phases [2]. The location of these refugia were purportedly in West, East, South and South-Western Africa [1], [9], [10], [19], [20].

Phylogeographic studies that accurately reflect the pattern of spatial genetic variation require access to large sample collections distributed across the entire range of the target species. Perhaps because of the difficulty in obtaining samples over such a large geographic area, as well as the inaccessibility of remote areas in Africa, it is rare to find studies involving more than 200 samples or which cover the entire distribution of the model species. To improve our understanding of African biogeography, we studied the spatial genetic structure of the African buffalo (*Syncerus caffer*) at a continental scale. The African buffalo is a good model because, together with a few other large taxa such as *e.g.* the elephant and the giraffe, it is distributed throughout most of sub-Saharan Africa. The species is primarily found in savannas, but also occurs in other habitats, including clearings in the rainforest belt [21]–[23]. The African buffalo exhibits extreme morphological variability across its range, greater than most other African mammals, both in body size and weight, coloration and horn size [21]. As a result, it has previously been assigned to as many as 52 distinct subspecies divided across two species [21], [24]. This number has decreased considerably as our understanding of the species has increased. Today, two [25], three [21] or four [26], [27] subspecies are commonly recognized (Table 1). The forest buffalo (*S. c. nanus*), which occurs in the West and Central African rainforests, shows adaptation to forest life [22] having a smaller size, unobtrusive swept-back horns and a characteristic red to reddish brown color. The Cape buffalo (*S. c. caffer*) of the South-East African savannas is about twice the size of the forest buffalo, with large downward curved horns and a brownish to black color. The third and fourth subspecies–not recognized by all authorities–are the West African savanna buffalo (*S. c. brachyceros*) and the Central African savanna buffalo (*S. c. aequinoctialis*), which are more difficult to distinguish morphologically. They occupy the Sahelian savanna, characterized by a low net above ground primary production compared to the East and Southern African savannas (±15% lower) [28]. The two latter subspecies seem to be morphologically intermediate between the Cape and forest buffalo [29]. The evolutionary relationships between these four putative subspecies are unclear, having therefore led to the taxonomic controversy. This uncertainty is corroborated by the observation of intermediate phenotypes in contact zones between all four subspecies [21] and of at least one reported cross between *S. c. caffer* and *S. c. nanus* in captivity, these being the two subspecies with the more divergent morphological characteristics [30]. Moreover, differences in karyotypes between the *nanus* and the *caffer* phenotypes have been reported, with *S. c. caffer* having 52 chromosomes and *S. c. nanus* having 2n = 54 to 56 chromosomes [30]–[32]. The extreme morphological variability within the species therefore allows us to test the congruence between phylogenetic-based and morphological-based classification.

**Table 1:** Morphological characteristics, including weight in kilogram (kg), dress color, body length in centimetre (cm), width of horn and length of skull in millimetre (mm) of the four recognized subspecies of African buffalos (out of literature).

	*S. c. caffer*	*S. c. nanus*	*S. c. aequinoctialis*	*S. c. brachyceros*
**Weight (kg)**	700–900 [26], [74]	<320 [26], [74]	intermediate	intermediate
**Dress**	blackish [78]	reddish [74], [76]	brown/black [75]	brown/black [75]
**Body length (cm)**	<340 [26], [74]	<120 [26], [74]	intermediate	intermediate
**Width of horn at base (mm)**	224±21.5 [46]	128±9.75 [46]	212±17.2 [46]	166±18.3 [46]
**Length of skull (mm)**	503±17.9 [46]	426±10.7 [46]	500±18.2 [46]	455±22.9 [46]

Although several genetic studies on African buffalo have already been conducted, these were generally focused at smaller spatial scales, mainly in East and Southern Africa (*S. c. caffer*), using various molecular markers such as mitochondrial DNA, autosomal microsatellites, Y-chromosomal microsatellites and the histocompatibility complex (MHC) *DRB3* gene [33]–[44]. Partial exceptions include the studies by Van Hooft [36], [37], which include a few samples (n = 14) from Central Africa. They highlighted a clear differentiation between *S. c. caffer* and *S. c nanus*/*S. c. brachyceros*. No significant genetic differentiation was found between *S. c. nanus* and *S. c. brachyceros* [37], but these results were not conclusive due to a limited sample size. A truly pan-African genetic study of the African buffalo has not been undertaken yet.

In the present study, a detailed genetic analysis of the African buffalo across its geographic range was performed using mitochondrial *D-loop* (control region) sequences. The control region is the most variable part of the mammalian mtDNA genome [45]. This study provides an unprecedented sampling scheme for buffalo, representing 43 study localities in 17 countries. We combined newly derived sequences from faeces and tissue biopsies from South, West and Central Africa with published sequences from East and Southern Africa. A total of 255 West-Central and 511 East-Southern buffalo mtDNA sequences were analyzed. Sequence data were analyzed with regard to demographic changes, phylogeography and evolutionary history. Specifically, we aimed to I) test whether there is a correspondence between the distinct morphological phenotypes and the genetic lineages, and II) infer the phylogeographic history that has led to the observed distribution of genetic and phenotypic variation.

## Results

As the taxonomic status of the subspecies is still subject to controversy (see above), we defined each of the four putative subspecies as recognized by East [27] and Kingdon [26] and adopted by the IUCN (2004) as an ecophenotype, *i.e., nanus* ecophenotype (forest-dwelling buffalo from West Africa), *brachyceros* ecophenotype (savanna buffalo from West Africa), *aequinoctialis* ecophenotype (savanna buffalo from Central Africa) and *caffer* ecophenotype (Cape buffalo from East and Southern Africa).

### Population Differentiation

#### 1. Network analysis and genetic distance

**Figure 1:**
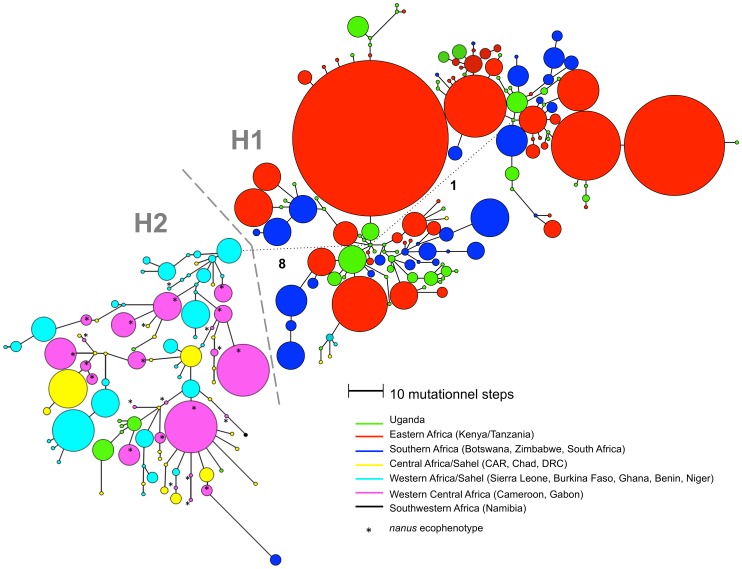
An unrooted minimum spanning tree (MST) of *Syncerus sp*. showing genetic relationship among *D-Loop* region haplotypes detected in this study. The sizes of circles are proportional to haplotype frequency and length of lines is proportional to the number of nucleotide substitutions separating the haplotypes, except for the dotted lines, where the numbers of mutational steps joining the circles are indicated above the connecting branches. Circles were colored according to geographical sample origin: Green/Red/Blue: H1 South and East Africa; Turquoise/Pink: H2 West Africa; Yellow: H2 Central Africa; Black: Namibia. Asterisks represent *nanus* ecophenotype samples. CAR: Central African Republic; DRC: Democratic Republic of the Congo.

Two lineages that made geographic sense were retrieved with the minimum spanning network (H1 and H2; Figure 1), following a South-Eastern/West-Central separation, with eight mutational steps separating the two lineages. Within these lineages, no finer geographic structure was evident, with samples from the various localities being paraphyletic. The first lineage (H1) is composed of South-Eastern (SE) African populations and contains 143 haplotypes, mainly of the *S. c. caffer* ecophenotype (136 haplotypes). This predominantly South-Eastern lineage also includes seven haplotypes of West-Central (WC) African origin. With the exception of the Namibian samples, haplotypes from Southern Africa (Zimbabwe, Botswana and South Africa) occupied the tip position in the network. About 45% of the Ugandan haplotypes occupied a central position in the network. The second geographic lineage (H2) consisted mainly of West-Central (WC) African samples, including samples from three ecophenotypes: *S. c. brachyceros*, *S. c. nanus* and *S. c. aequinoctialis*. These ecophenotypes were not monophyletic and were distributed throughout the H2 lineage. H2 included 94 distinct haplotypes, including seven of SE African origin.

The minimum spanning network is characterized by a clear star-like topology for the H1 lineage, and there is a tendency toward the same pattern for the H2 lineage. Distances separating SE African haplotypes were less than among the WC African haplotypes, reflecting the relatively low nucleotide diversity observed in SE Africa. Within each lineage, there was a low structural per country affinity of haplotypes.

#### 2. Genetic variation between populations and between subspecies

At the continental scale, African buffalo display high levels of genetic diversity, as reflected by both haplotype and nucleotide diversities (*h = *0.98, *π* = 5.50%; Table 2). As suggested by our previous results, a partition within two lineages at the continental scale has been revealed, with a high and significant proportion of the variation accounted for by the between-lineage component (*F_CT_ = *0.42). At the population level, low overall sequence divergence among the populations within each lineage was observed, ranging from 0.1% to 1.3% in H1 and from 0.1% to 2.9% in H2. According to the pairwise *F_ST_* values (Table 3), lower *F_ST_* values between populations within each lineage (SE *vs* WC) can be observed, compared to higher *F_ST_* values between populations between each of the lineages. Fixation indexes between populations between lineages (*F_ST_*) were estimated to be 0.52, and among populations within lineages (*F_SC_*) to be 0.17 (Table 4). This corroborates the absence of structure observed within lineages in the network and evolutionary trees. Moreover, pairwise *F_ST_* values between the *brachyceros*/*aequinoctialis* ecophenotypes and the *nanus* ecophenotype of Western Africa were quite low, ranging from 0.02 to 0.12, which is lower than between most population pairs within these ecophenotypes (Table 3 and 5).

**Table 2:** Genetic diversity and demographic test for both genetic lineages (H1 and H2), as well as for each ecophenotypes.

	*h*	*h* SD	*h p-value*	*π*	*π* SD	*π* *p-value*	Fu’s *Fs*	Fu’s *Fs* *p-value*	*τ*	*r*	*r* *p-value*	SSD	*SSD* *p-value*
**H1 (** ***i.e.,*** ** Ecophenotype ** ***caffer*** **)**	0.980	0.002	0.001	4.58	0.001	0.001	−24.016	*0.003*	6	0.003	*0.850*	0.001	0.670
**H2 (** ***i.e.,*** ** three other** **ecophenotypes)**	0.981	0.002	0.001	6.42	0.002	0.001	−23.902	*0.001*	13	0.005	*0.170*	0.002	0.190
**Ecophenotype ** ***aequinoctialis***	0.997	0.010	0.001	6.95	0.004	0.001							
**Ecophenotype ** ***nanus***	0.890	0.001	0.001	5.77	0.001	0.001							
**Ecophenotype ** ***brachyceros***	0.961	0.007	0.001	5.78	0.002	0.001							

*h*: haplotype diversity; *π*: nucleotide diversity expressed in percentage; SD: standard deviation;*τ:* expansion times*; r:* Harpending’s raggedness index; SSD: sum of squared deviations using ARLEQUIN and DNaSP software.

**Table 3:** Population pairwise *F_ST_* between the twenty-eight populations significantly differentiated calculated with ARLEQUIN software.

	1	2	3	4	5	6	7	8	9	10	11	12	13	14	15	16	17	18	19	20	21	22	23	24	25	26	27	28
1	**/**	***	***	***	***	***	ns	***	***	***	*	***	***	***	***	***	***	***	***	***	***	***	***	***	***	***	***	***
2	***0.39***	**/**	***	ns	***	***	ns	***	***	ns	*	***	***	***	***	***	***	***	***	***	***	***	***	***	***	***	***	***
3	***0.38***	***0.19***	**/**	*	***	***	*	***	***	***	***	***	***	***	***	***	***	***	***	***	***	***	***	***	***	***	***	***
4	***0.47***	***0.66***	***0.24***	**/**	ns	ns	ns	ns	ns	ns	ns	***	***	***	***	***	***	*	***	***	*	***	***	***	***	***	***	***
5	***0.22***	***0.11***	***0.14***	***0.01***	**/**	***	ns	***	***	ns	ns	***	***	***	***	***	***	***	***	***	***	***	***	***	***	***	***	***
6	0.30	0.22	0.27	0.10	0.13	**/**	*	***	*	***	***	***	***	***	***	***	***	***	***	***	***	***	***	***	***	***	***	***
7	0.07	0.22	0.20	0.10	0.01	0.16	**/**	*	ns	ns	ns	***	***	***	***	*	***	***	***	***	***	***	***	***	***	***	***	***
8	0.27	0.22	0.26	0.01	0.10	0.06	0.10	**/**	ns	***	***	***	***	***	***	***	***	***	***	***	***	***	***	***	***	***	***	***
9	0.26	0.23	0.28	0.06	0.08	0.08	0.08	0.04	**/**	*	***	***	***	***	***	***	***	***	***	***	***	***	***	***	***	***	***	***
10	0.29	0.07	0.22	0.02	0.02	0.13	0.05	0.14	0.12	**/**	ns	***	***	***	***	***	***	***	***	***	***	***	***	***	***	***	***	***
11	0.15	0.17	0.17	0.08	0.02	0.16	0.08	0.13	0.13	0.04	**/**	***	***	***	***	***	***	***	***	***	***	***	***	***	***	***	***	***
12	0.63	0.54	0.58	0.43	0.46	0.51	0.54	0.45	0.49	0.49	0.50	**/**	***	*	***	***	***	***	*	***	***	***	ns	***	***	***	***	***
13	0.70	0.57	0.60	0.53	0.48	0.52	0.64	0.45	0.52	0.55	0.55	0.11	**/**	*	***	*	*	*	ns	*	***	***	ns	***	***	***	***	***
14	0.63	0.50	0.54	0.35	0.42	0.46	0.50	0.39	0.45	0.44	0.45	0.04	0.04	**/**	***	ns	***	*	ns	***	***	***	*	***	*	***	***	*
15	0.61	0.49	0.54	0.37	0.40	0.46	0.49	0.40	0.45	0.43	0.45	0.05	0.18	0.09	**/**	***	***	***	***	***	***	***	*	***	***	***	***	***
16	0.70	0.54	0.58	0.46	0.45	0.48	0.61	0.40	0.49	0.50	0.50	0.17	0.08	0.06	0.22	**/**	*	ns	ns	nsns	ns	***	***	***	*	***	***	ns
17	0.68	0.56	0.60	0.46	0.47	0.51	0.59	0.43	0.50	0.52	0.52	0.10	0.08	0.06	0.17	0.06	**/**	***	ns	***	***	***	*	***	***	***	***	ns
18	0.67	0.52	0.55	0.44	0.42	0.47	0.56	0.40	0.47	0.46	0.47	0.13	0.15	0.08	0.20	0.13	0.12	**/**	ns	*	*	*	ns	***	ns	***	***	*
19	0.65	0.54	0.58	0.43	0.45	0.49	0.54	0.41	0.48	0.49	0.49	0.06	0.02	0.02	0.13	0.04	0.02	0.04	**/**	*	***	*	ns	***	ns	ns	***	***
20	0.64	0.56	0.59	0.44	0.48	0.52	0.55	0.44	0.50	0.52	0.53	0.15	0.05	0.07	0.17	0.02	0.10	0.11	0.05	**/**	***	***	*	***	***	***	***	*
21	0.60	0.21	0.54	0.38	0.43	0.46	0.50	0.40	0.45	0.46	0.46	0.21	0.12	0.14	0.18	0.05	0.14	0.18	0.11	0.06	**/**	***	***	***	***	***	***	*
22	0.72	0.64	0.66	0.62	0.55	0.59	0.67	0.52	0.59	0.63	0.62	0.17	0.12	0.16	0.30	0.23	0.14	0.09	0.06	0.18	0.26	**/**	ns	***	***	***	***	***
23	0.65	0.51	0.54	0.40	0.41	0.46	0.51	0.39	0.45	0.43	0.43	0.08	0.06	0.05	0.15	0.15	0.08	0.03	0.01	0.09	0.15	0.01	**/**	*	*	***	***	***
24	0.64	0.52	0.57	0.40	0.45	0.50	0.54	0.44	0.49	0.46	0.49	0.06	0.20	0.08	0.09	0.24	0.16	0.18	0.11	0.20	0.24	0.25	0.12	**/**	***	ns	***	***
25	0.61	0.51	0.56	0.38	0.44	0.48	0.50	0.42	0.46	0.46	0.47	0.09	0.13	0.04	0.14	0.09	0.08	0.02	0.03	0.11	0.17	0.13	0.07	0.10	**/**	***	***	***
26	0.60	0.51	0.55	0.39	0.44	0.48	0.50	0.42	0.46	0.46	0.46	0.05	0.09	0.07	0.09	0.16	0.12	0.10	0.06	0.15	0.19	0.17	0.08	0.03	0.04	**/**	***	***
27	0.73	0.61	0.63	0.58	0.52	0.56	0.67	0.52	0.59	0.57	0.59	0.33	0.52	0.37	0.32	0.49	0.43	0.45	0.41	0.42	0.42	0.56	0.44	0.29	0.28	0.25	**/**	***
28	0.68	0.52	0.57	0.38	0.43	0.47	0.56	0.39	0.47	0.46	0.48	0.17	0.15	0.06	0.19	0.01	0.08	0.16	0.08	0.06	0.09	0.30	0.19	0.22	0.11	0.09	0.63	**/**

The significance level is shown above the diagonal, *indicates *P*<0.05, **indicates *P*<0.005, ***indicates *P*<0.0005; ns, non-significant. From line 1 to 11: H2 lineage (*S. c. nanus, S. c. brachyceros and S. c. aequinoctialis*); from line 12 to 28: H1 lineage (*S. c. caffer*); bold/italic written value: ecophenotype *nanus*. 1: Campo ma’an; 2: Lope; 3: Gamba; 4: Ngoto Forest; 5: Benoue; 6: Mole; 7: Arly, Pama, Singou; 8: Pendjari; 9: W; 10: Zakouma; 11: Bamingui-Bangoran; 12: Queen Elizabeth; 13: Lake Mburo; 14: Murchison falls; 15: Kidepo valley; 16: Laikipia; 17: Amboseli; 18: Nairobi; 19: Tsavo; 20: Masai Mara- Serengeti- Maswa; 21: Nakuru; 22: Arusha; 23: Kizigo; 24: Hwange-Chobe; 25: Kruger; 26: Gonarezhou; 27: Hluhluwe-Imfolozi; 28: Mount Elgon.

**Table 4:** Fixation indices as estimated from hierarchical AMOVAs in ARLEQUIN software. This analysis was computed with the twenty-eight populations distributed within the two geographical lineages (H1 and H2) (*p*<0.001) (*F_SC_*: among populations within lineages; *F_ST_*: among populations among lineages; *F_CT_*: among both lineages).

	*F*-statistics
***F_SC_***	0.17
***F_ST_***	0.52
***F_CT_***	0.42

**Table 5:** Pairwise *F_ST_* computation between ecophenotypes.

	Ecophenotype *nanus*	Ecophenotype *brachyceros*	Ecophenotype *aequinoctialis*
**Ecophenotype ** ***brachyceros***	0.12		
**Ecophenotype ** ***aequinoctialis***	0.02	0.08	
**Ecophenotype ** ***caffer***	0.51	0.45	0.44

Nucleotide diversity is lower for H1 (4.6%) than for H2 (6.4%), except for the Campo-Ma’an population (Table 6 and 2). The highest nucleotide diversity was observed in the *aequinoctialis* ecophenotype populations (7%), being on average 1.4 times higher than in the *caffer* ecophenotype populations. The largest distances, up to 2.9% (higher *F_ST_* = 0.47 (Table 3)), were found among forest buffalo populations (*S. c. nanus*). The *nanus* ecophenotype also displays the lowest haplotype diversity, being on average 1.3 times lower than in the other populations.

**Table 6:** Sample table summarizing their origin, number per park, subspecies affiliation based on morphological data, number of haplotypes, and haplotype and nucleotide diversities with their associated standard deviation estimated with ARLEQUIN and DNaSP software.

Country	TotN_ind_	Locality within each country	spp	LocalN_ind_	Local N_hap_	*h*	SD	*π* (%)	SD
**Sierra Leone**	1	Gola Forest	A	1	1	/	/	/	/
**Ghana**	39	Mole	B	37	13	0.878	0.037	5.42	0.28
		Kpetsu	A	2	2	/	/	/	/
**Burkina Faso**	5	Arly, Pama, Singou	B	5	4	0.900	0.161	6.40	1.58
**Benin**	41	Pendjari	B	41	19	0.931	0.021	5.72	0.32
**Niger**	21	W	B	21	14	0.948	0.031	5.04	0.46
**Cameroon**	37	Benoue	A,B	37	15	0.938	0.016	5.98	0.28
**Gabon**	76	Campo ma’an	A	21	3	0.600	0.080	2.77	0.43
		Gamba	A	30	5	0.667	0.063	4.85	0.52
		Lope	A	25	6	0.760	0.073	5.07	0.45
**CAR**	25	Ngoto Forest	A,C	4	4	1.000	0.002	7.95	0.29
		St-Floris	C	3	3	/	/	/	/
		Bangoran	C	10	10	1.000	0.001	7.63	0.25
		Koukourou	C	2	2	/	/	/	/
		Sangha	C	1	1	/	/	/	/
		Ouadda	C	2	2	/	/	/	/
		Bria	C	1	1	/	/	/	/
		Ndji River	C	1	1	/	/	/	/
		Mbari	C	1	1	/	/	/	/
**Chad**	10	Zakouma	C	10	9	0.978	0.046	7.08	0.51
**DRC**	1	Garamba	A	1	1	/	/	/	/
**Uganda**	134	Queen Elizabeth	D	60	24	0.948	0.012	5.52	0.37
		Lake Mburo	D	22	17	0.965	0.034	2.36	0.52
		Murchison falls	D	21	19	0.990	0.018	4.82	0.55
		Kidepo Valley	D	19	14	0.953	0.036	4.30	0.47
		Mount Elgon	D	12	8	0.909	0.025	3.46	0.34
**Kenya**	127	Laikipia	D	10	6	0.844	0.103	2.55	0.59
		Amboseli	D	20	10	0.863	0.063	3.29	0.44
		Nairobi	D	10	4	0.711	0.117	3.25	0.56
		Tsavo	D	24	11	0.909	0.032	3.61	0.45
		Masai Mara	D	28	11	0.902	0.029	3.32	0.43
		Nakuru	D	35	9	0.840	0.037	4.23	0.85
**Tanzania**	119	Serengeti	D	37	15	0.899	0.033	3.66	0.36
		Maswa	D	22	10	0.870	0.052	3.96	0.61
		Arusha	D	48	11	0.735	0.064	2.34	0.37
		Kizigo	D	9	5	0.806	0.120	3.80	1.02
		Selous	D	1	1	/	/	/	/
		Unknown origin	D	2	2	/	/	/	/
**Zimbabwe**	59	Hwange	D	16	9	0.883	0.062	5.08	0.52
		Gonarezhou	D	43	19	0.955	0.038	4.59	0.55
**Botswana**	11	Chobe	D	11	10	0.982	0.046	4.53	0.58
**South Africa**	60	Kruger	D	41	15	0.929	0.017	4.53	0.33
		Hluhluwe-Imfolozi	D	19	2	0.515	0.096	3.17	0.56
**Namibia**	1	Mother from Okahandja	E	1	1	/	/	/	/
**AVERAGE**						0.871	0.051	4.525	0.511

The nineteen samples excluded from the analysis are not quoted in this table (A *S. c. nanus*; B *S. c. brachyceros*; C *S. c. aequinoctialis*; D *S. c. caffer*; E Unknown affiliation; spp: subspecies; *h*: Haplotype diversity; *π*: nucleotide diversity expressed in percentage; CAR: Central African Republic; DRC: Democratic Republic of the Congo). The samples from the Benoue National Park and the Ngoto forest were morphologically intermediate between the *S. c. nanus* and the *S. c. brachyceros*/*S. c. aequinoctialis* subspecies respectively, indicated by the both capital letter.

### Demographic Trends

**Figure 2:**
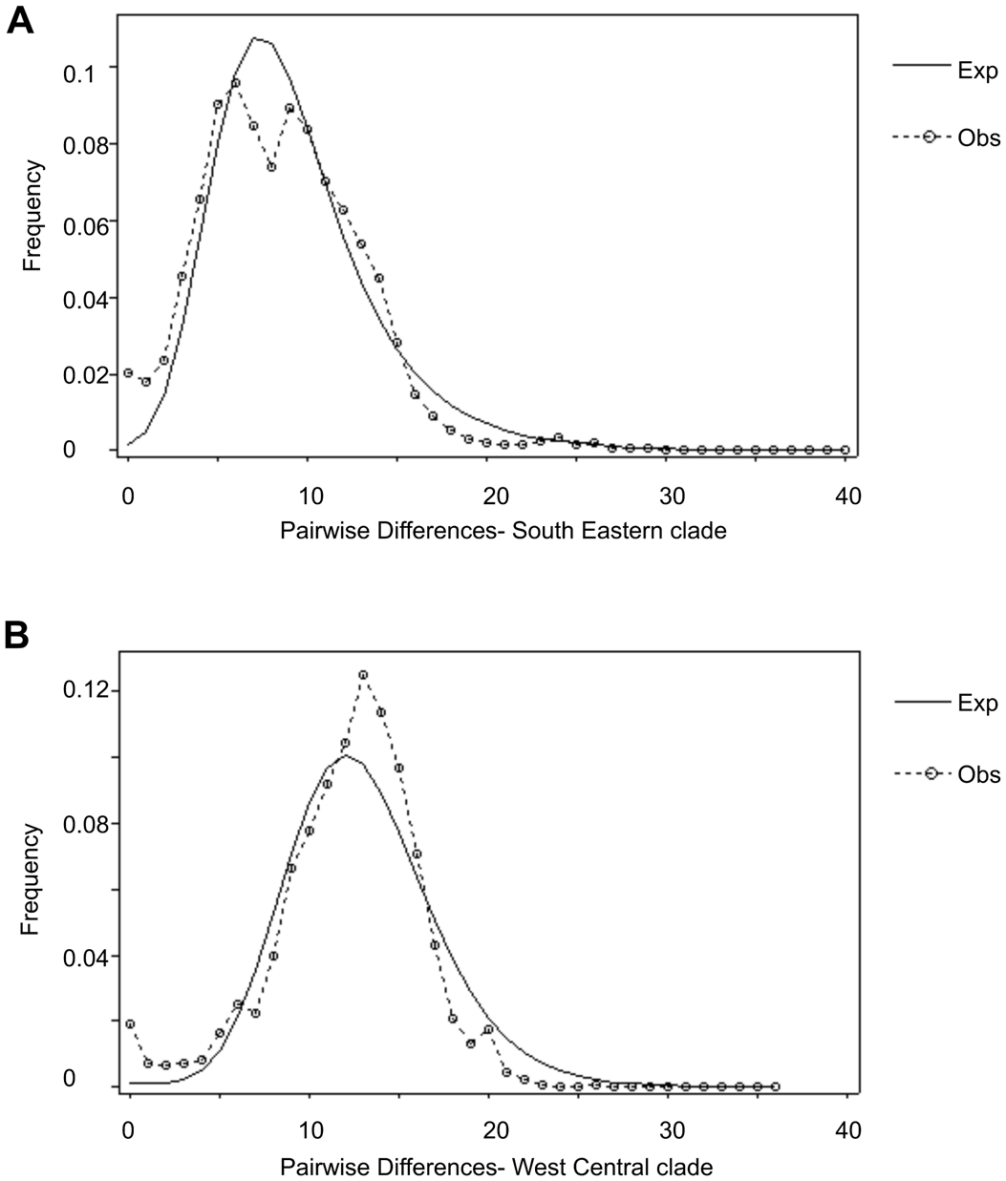
Mismatch distribution analysis including whole samples from the four subspecies, with observed (dotted line) and expected (thin line) mismatch from segregating sites of the aligned sequences of the *D-Loop* gene computed under the sudden expansion model performed with the ARLEQUIN software. A South-Eastern lineage; B West-Central lineage.

The mismatch distribution analysis showed a predominantly unimodal pattern for both lineages (Figure 2). In addition, Fu’s *Fs* values as well as the raggedness index were significantly negative (Table 2) (H1: *p = *0.007; H2: *p = *0.001) which suggests a demographic expansion for both lineages. According to the *τ* values (*τ*_H1_ = 6; *τ*_H2_ = 13), demographic expansion for H1 was estimated as starting approximately 48 000 YBP (Year Before Present), and H2 approximately 104 000 YBP.

### Coalescence Based Analyses

The phylogenetic analyses performed with the program BEAST confirmed the clear separation into two distinct geographical lineages, namely H1 and H2, as revealed by the minimum spanning network.

**Figure 3:**
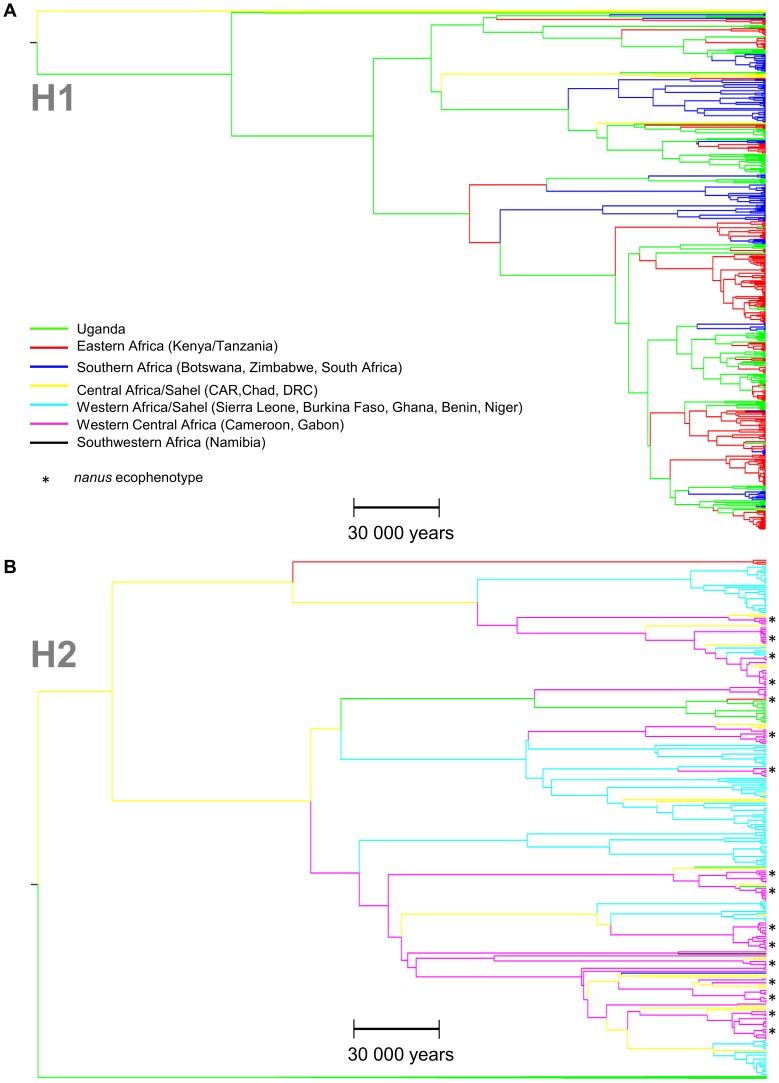
Bayesian phylogeographic trees of the *D-Loop* sequences reconstructed with BEAST software showing the inferred geographical location of each node in the buffalo phylogeny. Node locations are color coded (branches leading to each node) according to geographical sample origin: Green/Red/Blue: H1 South and East Africa; Turquoise/Pink: H2 West Africa; Yellow: H2 Central Africa; Black: Namibia. Scale bar shows time in years. Asterisks represent *nanus* ecophenotype samples. A H1 lineage; B H2 lineage. CAR: Central African Republic; DRC: Democratic Republic of the Congo.

**Figure 4:**
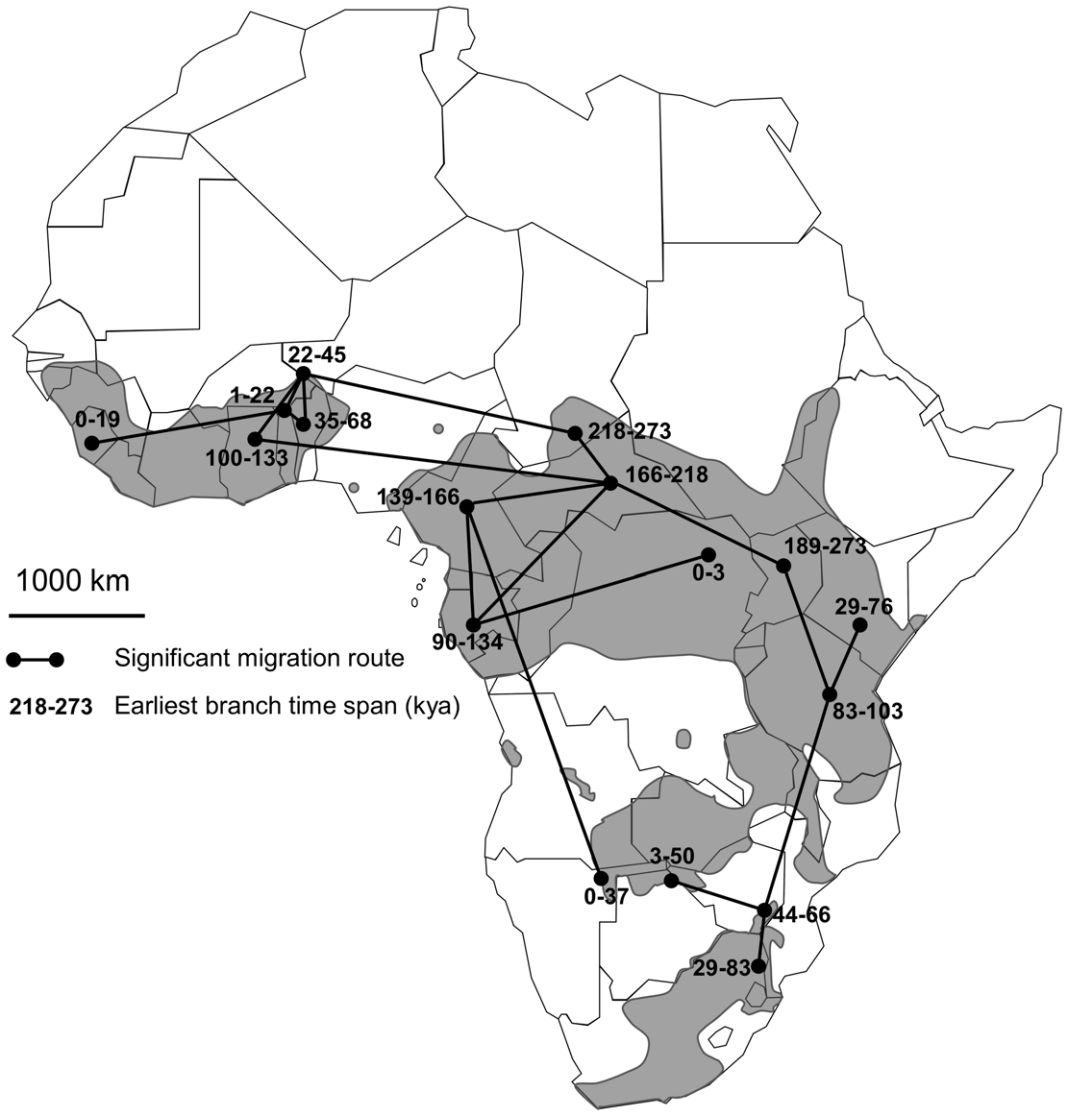
Map of the African continent showing the seventeen historical migration rates between sampled localities supported by a Bayes factor >3. Grey shape on the map represent the actual distribution of the African buffalo after IUCN’s Antelope Specialist Group, 2008. Numbers on the map indicate the median time endpoints over all BEAST trees of the earliest branch with a given locality state. Hence, it provides an estimate of the earliest migration into each locality.

For a better visual clarity of the BEAST results, the global tree is presented as two subtrees corresponding to the lineages H1 and H2 (Figure 3A and 3B). Although the root location could not be inferred with high confidence, results favored Chad/CAR/Uganda as the root location (posterior probability = 0.40 compared to prior probability 0.18). Uganda was the most probable location for many of the older internal nodes in H1, with high posterior probability (>0.5). In contrast, H2 showed comparatively low support for node locations, although the posterior probabilities for most of the best-supported locations were higher than 0.20 (Chad, Cameroon and CAR: collective posterior probability of 0.20+0.20+0.19 = 0.59, compared to prior probability = 0.18). As can be seen in the network (Figure 1), although H1 and H2 mainly consist of samples from South-Eastern and Western Africa respectively, there were a number of exceptions to this. In the tree, a separate lineage of 13 East African samples (of which twelve were Ugandan) was observed in the H2 lineage. Out of all 120 possible between-state diffusion rates, 17 migration rates had a Bayes factor of more than 3 (Figure 4).

**Figure 5:**
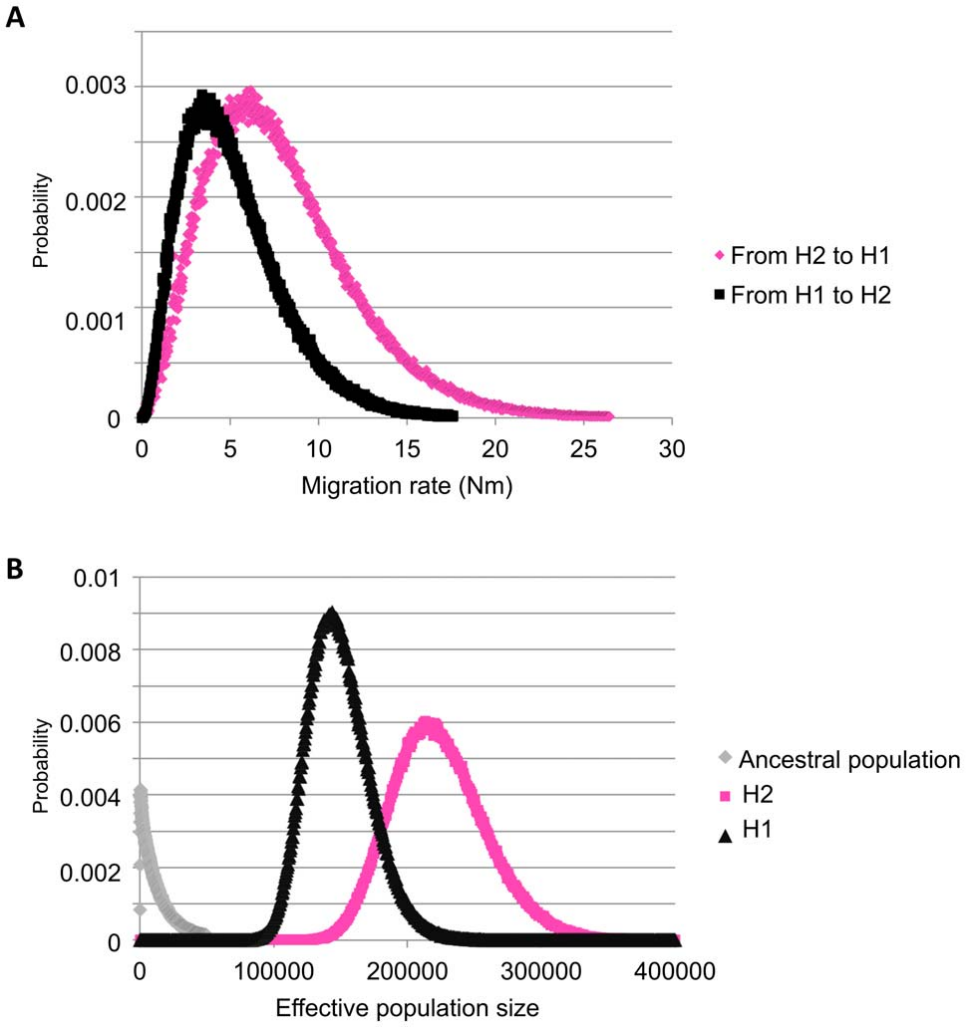
Plots of the posterior probability distribution of parameters estimated from the isolation-with-migration model performed with the IM software. A Posterior distribution for migration estimates of the directional migration rates from H1 to H2 (in black) and otherwise (in pink); B Posterior distribution of estimates of the population sizes for the ancestral (in grey), H1 (in black) and H2 (in pink) populations.

The divergence time between the H1 and the H2 lineages under the IM model was estimated at 193 kyr (95% CI: 145 kyr–449 kyr). With the BEAST reconstruction, the divergence time at the root of the tree was estimated at 273 kyr, with a divergence between a Ugandan and a Chad lineage, situated within the confidence interval of the divergence time estimated considering gene flow between both lineages over time. This gene flow was estimated at around five female migrants per generation (95% CI 1–17) with IM. Gene flow did not appear to be directional (*p*≥0.49) (Figure 5A). The effective population size was assessed to be larger for H2 (215 000, 95% CI: 160 000–292 000) than for H1 (149 000, 95% CI: 109 000–205 000) (*p* = 0.05). The ancestral effective population size based on the same dataset (850, 95% CI: 245–27 000) was significantly smaller than the current effective size (*p*<0.00001), indicative of an important population expansion event (Figure 5B). A relatively high negative correlation was observed between divergence time and ancestral population size (Pearson r = −0.54), indicating that the IM program had difficulties estimating these two parameters independently. All other pairwise correlations between parameters were estimated to be between −0.34 and 0.29. The split parameter indicates that it is possible that only a small fraction (fraction: 0.14, 95% CI: 0.12–0.98) of the ancestral population founded the extant metapopulation in SE Africa (*i.e., S. c. caffer*).

## Discussion

### Continental Phylogeographic Structure and Taxonomic Status of the Buffalo

Our study, based on the mtDNA genetic structure points to a genetic discontinuity between the WC (mainly lineage H2) and the SE (mainly lineage H1) populations (*F_CT_ = *0.42). Van Hooft [37] found similar results on the basis of mitochondrial and Y-chromosomal markers, with a smaller sampling from WC Africa (14 samples). These lineages or haplogroups are not equivalent to populations, but the clear separation between them suggests that they are the results of a significant divergence and hence represent Management Units (or MUs; Moritz 1994). The lineages correspond to the two subspecies distinguished by all authorities: *S. c. caffer* for the SE populations, and *S. c. nanus* for the WC populations. According to these genetic results, *S. c. nanus* would include *S. c. brachyceros* and *S. c. aequinoctialis* subspecies following standard nomenclature rules (S. c. nanus Boddaert 1785, S. c. brachyceros Gray 1837 and S. c. aequinoctialis Blyth 1866). This is further substantiated by the low amount of genetic differentiation between *S. c. brachyceros*, *S. c. aequinoctialis* and *S. c. nanus*. Although inferences based on DNA data are somewhat hampered because of the time required for complete lineage sorting to occur, our data unequivocally show that the separation between *S. c. brachyceros*, *S. c. nanus* and *S. c. aequinoctialis* is not taxonomically equivalent to the split between all of these and *S. c. caffer*. We note that these are preliminary genetic results as they are based exclusively on single-locus mtDNA. No final conclusion can be drawn concerning the taxonomic status of these subspecies before using complementary nuclear markers.

The absence of genetic structure within H2 based on the analysis of the mitochondrial DNA -contrasting with extreme phenotypic variability- also suggests that buffalo may rapidly adapt (in evolutionary terms) to different ecological conditions, with ecophenotypes not being reproductively isolated. Finer scale resolution of the connectivity between the three ecophenotypes of WC Africa should be investigated using more sensible molecular markers as microsatellites or single nucleotide polymorphism. Besides, based on our results, the fourth species described in Groves and Grubb [46], *Syncerus mathewsi*, a population restricted to the forested mountainous area of Virunga volcanoes, would correspond to an intermediate between the *nanus* and the *caffer* ecophenotypes (contact region close to the Rift Valley), with morphological intermediate characteristics, as also proposed by Grubb [47] himself. Further investigations using neutral nuclear markers within the contact region would permit clarification of this last assumption.

Although the morphology, behavior and respective habitats of *S. c. caffer* and S. c. nanus are very different, the amount of genetic differentiation is typical of that of subspecies, especially when compared to the range of *F_CT_* values observed for other large African bovids (Table 7). The most parsimonious explanation for this pattern is that an ancient allopatric separation between an Eastern and a Western population formed the two observed lineages. This is further supported by earlier findings based on Y-chromosomal microsatellite loci, which indicated that while *S. c. caffer* was monomorphic for a single allele, the same allele was not present in *S. c. nanus* [37]. Nevertheless, gene flow between those two lineages, even if low, was indicated by a few haplotype infidelities in the network and the phylogenetic analyses. This gene flow was estimated to be in the order of five mitochondrial genomes per generation since these lineages diverged. The difference in chromosome number between the *nanus* and the *caffer* ecophenotype, due to a Robertsonian fusion of two acrocentric pairs (*S. c. caffer*: 52 and *nanus* ecophenotype: 54–56 chromosomes) [30]–[32], may have also contributed to a low gene flow by decreasing hybrid fertility [48] and may be a consequence of their divergence in allopatry. Negative effects of the variation of chromosome number on fertility have been observed in mice, cattle and humans [49], [50].

**Table 7:** Maximum fixation indices (*F_CT_*) per species for mtDNA between subspecies or genetic lineages reported in various Bovinae and African mammals.

Species	*F_CT_* mtDNA	Reference
**Giraffe, ** ***Giraffa camelopardalis***	0.75	[77]
**Topi, ** ***Damaliscus lunatus***	0.70	[1]
**Warthog, ** ***Phacochoerus africanus***	0.69	[2]
**Hartebeest, ** ***Alcelaphus buselaphus***	0.68	[1]
**Greater kudu, ** ***Tragelaphus strepsiceros***	0.57	[3]
**Wildebeest, ** ***Connochaetes taurinus***	0.54	[1]
**Roan antelope, ** ***Hippotragus equinus***	0.49	[9]
**Kob, ** ***Kobus kob***	0.49	[6]
**African buffalo, ** ***Syncerus caffer***	0.42	[This study]
**Impala, ** ***Aepyceros melampus***	0.33	[3]
**Bonobo, ** ***Pan paniscus***	0.31	[78]
**Savanna elephant, ** ***Loxodonta africana***	0.29	[11]
**Hippotamus, ** ***Hippopotamus amphibius***	0.10	[79]

The separation into two lineages, distributed in WC and SE Africa respectively, is a pattern observed in many other savanna African mammal species, as for example within the roan antelope (*Hippotragus equinus*) or the bushbuck (*Tragelaphus scriptus*) [9], [12]. Convergent phylogeographic patterns could indicate the existence of common African refugia for various African savanna species during climatic oscillations [18], proposed to be located in West, East, Southern and South-West Africa [51]. Buffalo populations in Uganda and in the Central African Republic displayed the highest genetic diversity found in the species and were the most likely candidates for the tree root location (Figure 3), suggesting that these are good candidate areas for historic refugia within this species. As the two lineages are found around this region, it may also be considered as a hybrid or overlapping zone between populations of the two lineages, explaining the higher genetic diversity observed. The same phylogeographical pattern is observed within the kob (Kobus kob- based on nuclear DNA and mtDNA) with an overlapping region located around Northern Uganda [7]. It was proposed based on divergent phenotype and life-history adaptations of the kob subspecies that populations went isolated within refugia in West and East Africa during the Pleistocene, with subsequent dispersal leading to secondary contact and hybridization between lineages around the present-day Uganda. Similar refugia location were also proposed for the hartebeest (Alcelaphus buselaphus- based on mtDNA), the topi (Damaliscus lunatus- based on mtDNA) and the roan antelopes (Hippotragus equinus based on both nuclear DNA and mtDNA) [1], [6], [7], [9]. Based on the concordant pattern of intraspecific structure in African mammals, we propose that the two lineages division observed in different savanna species has arisen as a consequence of Pleistocene climate oscillations in the absence of an obvious present-day geographical barrier. More arid conditions observed during glacial periods would have promoted isolation of populations in refugia, with expansion during interglacial wet periods [8], with secondary contact and hybridization between lineages when overlapping occured. We thus propose that the African buffalo survived unfavorable periods during the Pleistocene in at least one refuge located in Western and one refuge located in Eastern Africa, with overlapping around the present-day Uganda.

Even if the buffalo would show strong philopatric behavior [21], [52], [53], its high nucleotide diversity and low differentiation between populations suggest a capability to disperse widely over evolutionary time scales. The combination of a relatively large effective population size and the possible ancestral nature of the H2 lineage as discussed hereafter may explain its higher nucleotide diversity. Furthermore, it is interesting to note that the overall nucleotide diversity reaches 6%, which, except for the Grant’s gazelle and the bushbuck, is high in comparison to other large African mammals, even if buffalo populations are reported to have declined in numbers due to outbreak of rinderpest during the last century (elephant (*Loxodonta africana*), 1.4%; bushbuck (*Tragelaphus scriptus*), 11.7%; roan antelope (*Hippotragus equinus*), 1.6%; waterbuck (*Kobus ellipsiprymnus*), 4.1%; impala (*Aepyceros melampus*), 2.8%; Grant’s gazelle (*Nanger granti*), 10.9%) [9], [12], [54]–[57]. Same high level of genetic variation was found in previous studies investigating the impact of such severe population bottlenecks on the genetic variability of buffalo populations, indicating that rinderpest pandemic had very little impact on the genetic diversity of the African buffalo in terms of allelic or haplotype diversity [35], [43], [55]. The *nanus* ecophenotype populations are in comparison relatively small and isolated, as indicated by low haplotype diversities and high between-population genetic distances. Pairwise *F*_ST_ values between the *nanus* ecophenotype populations were about three times as large as those between populations within the other ecophenotypes (see bold/italic written *F_ST_* value in Table 3), indicating that forest buffalo populations show signs of fragmentation and of genetic drift, reflecting the importance of rainforest as a biogeographical barrier to gene flow. Observations of forest-dwelling buffalo (*nanus* ecophenotype) confirm our assumption [21], [58], [59]. These studies showed a high correlation between forest buffalo presence and clearings, necessary for feeding and for water accessibility [23]. This dependence on open places has been identified as a limiting factor in movement of individuals. Our results support the fact that rainforest acts as a major biogeographic barrier limiting gene flow.

### Evolutionary History and Demographic Trends

According to the IM analysis, although this result was not significant, the split parameter suggests that the *S. c. caffer* subspecies originates from the isolation of a subpopulation of the ancestral population. Fossil records from the late Pleistocene are in agreement with this. Indeed, the close resemblance of the Pleistocene-dated fossils to the actual West-Central African buffalo suggests that the H2 population existed before the H1 population [52], [60]. Our results therefore suggest a recent origin of the Cape buffalo (*S. c. caffer*), which possibly derived from a stock of savanna buffalo originating from West-Central Africa (H2 lineage) [52].

The coalescent approach with migration suggested a Middle to Late Pleistocene divergence (150–300 kyr), which is contemporaneous with previous studies of other African species [1], [6]. It should be noted that we are restricted to making inferences going back to the MRCA (the ‘mitochondrial Eve’) of the buffalo mtDNA, which is most likely much younger than the species itself, unfolding only part of the buffalo history.

We also found evidence under the IM model of an important expansion in both lineages since their Pleistocene divergence, which is supported by the star-like topology of the haplotype network of the H1 lineage and the negative Fu’s *Fs* index for both lineages. This is in agreement with two earlier studies based on *S. c. caffer,* using an alternative coalescent approach with Bayesian skyline plots [61], [62]. The expansion time, calculated based on the *τ* values, was estimated as starting at approximately 48 000 YBP for the H1 lineage, consistent with a previous study on the Cape buffalo by Van Hooft [37]. Expansion time for the H2 lineage was estimated at approximately 104 000 YBP. The more recent expansion of the H1 compared to the H2 lineage could be related to the development of open grassland on the East-Southern part of the continent at the end of Pleistocene. Paleoclimatic indications support our assumption. Indeed, Eastern and Southern Africa experienced an extremely arid period between 135 and 90 kyr, far more severe than the conditions occurring during the Last Glacial Maximum (35–15 kyr). Aridity decreased progressively after 95 kyr, until it reached near modern conditions around 60 kyr [63]. This period approximately coincides with the expansion signal for the H1 lineage. The development of large savanna-type grasslands could have allowed southward colonization by providing suitable habitat for the buffalo populations [64], [65]. Van Hooft [37] also proposed that this expansion could have followed the extinction of a buffalo-like species, the giant long-horned buffalo (*Peloveris antiquus*), which dominated the African savanna until the late Pleistocene, as demonstrated by fossil data [52], [66], [67], a hypothesis also in agreement with our results.

Nevertheless, recent radiometric studies on fossil records support the presence of buffalo in Southern Africa around 542 kyr (95% CI: 435–682 kyr) [68], which indicates that some *Syncerus*-like species occupied Southern Africa earlier than we could infer based on mtDNA. Our signal of expansion toward Southern Africa could thus be a signal of re-colonization, which supports the hypothesis of the importance of the refugia located in Eastern and Western Africa. Southern Africa could have witnessed multiple colonization-extinction events, following habitat suitability.

Lineage H2 on the other hand was inferred to have expanded earlier, at approximately 104 000 YBP. More varied and open habitats prevailed after 1.8 Ma in subtropical Africa [69], with more pronounced vegetal changes after 220 kyr associated with climatic shifts [70], [71]. Paleorecords support the probable persistence of a rainforest zonal belt in both dry and wet periods before 220 kyr, with large westward expansions of *Podocarpus* forest during favorable periods [70]. After 220 kyr, a more pronounced reduction of scattered occurrences of the rainforest was recorded. The development of savanna habitat in Western Africa, replacing forest habitat, could have promoted the expansion of the Western populations of savanna buffalo, whose expansion signal approximately coincides with the savanna development.

### Major Colonization Events and Migration Routes

The Eastern region around the present-day Uganda appears to have played a prominent role throughout the history of the H1 lineage. Many internal branches had high posterior probabilities for the geographical state of Uganda, and the earliest occurrence of a non-Ugandan branch in H1 was 83–103 kyr (Figure 3B), roughly 170 000 years after the divergence of H1 and H2. This supports an important conclusion: a primary refuge for *S. c. caffer* located in Eastern Africa appears to have played an important role in its history, when climatic conditions were unfavorable. Migration from a core Eastern refuge into other parts of SE Africa was found to have happened several times, as these other locations are positioned at multiple separate external branches, dated independently, on internal Ugandan branches, probably related to the climatic oscillation registered during Pleistocene. Further support is found in the network reconstruction, which reflects the same pattern, with Southern African haplotypes forming sub-groups positioned at tip positions, without being monophyletic. Our analysis of the quantification of the strength of connectivity between geographical states in the tree, which attempted to identify the historically most important migration routes, identified strong support for a Uganda-Tanzania and a Tanzania-Kenya link, but not for a Uganda-Kenya link. Thus, a central role of Tanzania in this Southern expansion was also highlighted.

The H2 lineage showed a less clear geographical pattern with an earlier geographical diversification from a core population in Chad, Cameroon or the Central African Republic (CAR) (*i.e.,* Western refuge). The genealogy of H2, with its longer branch lengths, indicates that H2 underwent a demographic expansion earlier than H1, a fact corroborated by the mismatch distribution profiles. For H2, Cameroon and Gabon hosting forest buffalo ecophenotypes form migratory cul-de-sacs connected to CAR, indicating that these populations are the result of a unique migration route from CAR, which is different from the migration route between CAR and the Western savanna buffalo populations. This is consistent with Figure 3A, where the same three Central African forest buffalo populations mainly occur together in lineages distinct from the Western savanna buffalo of Niger, Chad, Ghana and Benin. This interpretation suggests that the forest buffalo ecophenotype, rather than being the ancestor of all living African buffalo as Kingdon [26] proposed, may be an advanced form derived from the WC savanna ecophenotype. This is also supported by the fact that the forest ecophenotype appears to be non-monophyletic, indicating different separate migration route endpoints. Furthermore, the work of Bekhuis [23], based on the study of the diet of the forest buffalo in Cameroon, concluded that the ancestral niche of the buffalo ancestors more likely corresponds to a savanna-rainforest gradient or mosaic than to a true rainforest, in agreement with the observation that most Pleistocene buffalo fossils resemble *S. c. brachyceros* [72]. CAR was clearly an important link in the westward dispersal of buffalo from a presumed Central African origin, as evidenced by the high number of strongly supported rates between CAR and other H2 locations in addition to the inferred link between CAR and Uganda.

Interestingly, the one available sample from South-Western Africa (Namibia) was positioned well within H2. This shows an important migration connection between West-Central and West-Southern Africa, which has been proposed before [37] and is supported by morphological studies, reporting the existence of a dwarf-buffalo-like-population in Angola [21]. Nevertheless, as this deduction is based on only one sample from Antwerpen Zoo with mother from Okahandja [37, this study], it should be regarded as tentative and to be further investigated by increasing the sampling from this region.

In summary, the pattern in Figure 4 and Figure 3 suggests that the split between two major lineages of buffalo occurred in Western Africa, probably around the present-day CAR. One lineage (H1) apparently remained in Eastern Africa for a long time before expanding in population size and range, and the other (H2) expanded earlier along two separate routes into the African forest belt and the Western Sahel region, respectively. Overall, we propose the following phylogeographic scenario: the ancestor of all living buffalo lived in Western Africa. This ancestral population became separated (for unknown reasons) around 100–300 kyr into two isolated populations, one of which (ancestors of H2) started expanding westward at an early time, around 100 kyr or earlier. The other (ancestors of H1) retained its core population in Eastern Africa, probably being unable to colonize arid Southern Africa until about 50–80 kyr, where it expanded South and East, possibly after adapting to an arid savanna environment or after the decline of an obvious competitor, *Pelorovis antiquus* [52], [66], [67]. A recent colonization of Southern Africa by *S. c. caffer* is supported by the lack of true Cape buffalo characteristics, *i.e.,* the sweeping horns and pronounced horn boss, in all buffalo fossils from this region [52], [72]. This tentative phylogeographic scenario is in agreement with the wealth of fossil and molecular data currently available on the African buffalo.

### Conclusions

Our main finding is that African buffalo should be partitioned into two MUs (*i.e.,* Management Units as defined by Moritz [73]), putatively named the South-Eastern African buffalo (*S. c. caffer*) and the Western African buffalo (*S. c. nanus*), which has important implications for the conservation of the species. We found little genetic structure distinguishing the three morphologically distinct ecophenotypes of the Western African buffalo, hence we posit that these ecophenotypes merely represent rapid adaptations to local habitat variations without reproductive isolation. Our results also suggest that the forest buffalo ecophenotype may be an adapted form derived from the West-Central savanna ecophenotype. The more important phenotypic variability observed in West-Central Africa could be the result of an earlier origin of the Western lineage than of the Cape buffalo. More genetic clusters in the Western lineage that very recently diverged may also exist and could be identified using finer genetic markers. We have demonstrated that extensive sampling from the whole distribution of a species makes it possible to infer important aspects of historical migration, refugial areas and taxonomic subdivision.

## Materials and Methods

### Sample Collection and Laboratory Methods

The protocols for animal sampling used for our study did not induce pain or distress, according to the Animal Care Resource Guide, and thus correspond to USDA category C. Indeed, capture relied on the live capture technique of large mammals covered in the American Society Mammalogists Guidelines. Procedures were not more invasive than peripheral blood sampling or peripheral tissue sampling. Chemical immobilisation was only performed to facilitate the procedure. Animals were released in favourable condition that enabled them to avoid predators, seek shelter, and survive inclement weather.

**Figure 6:**
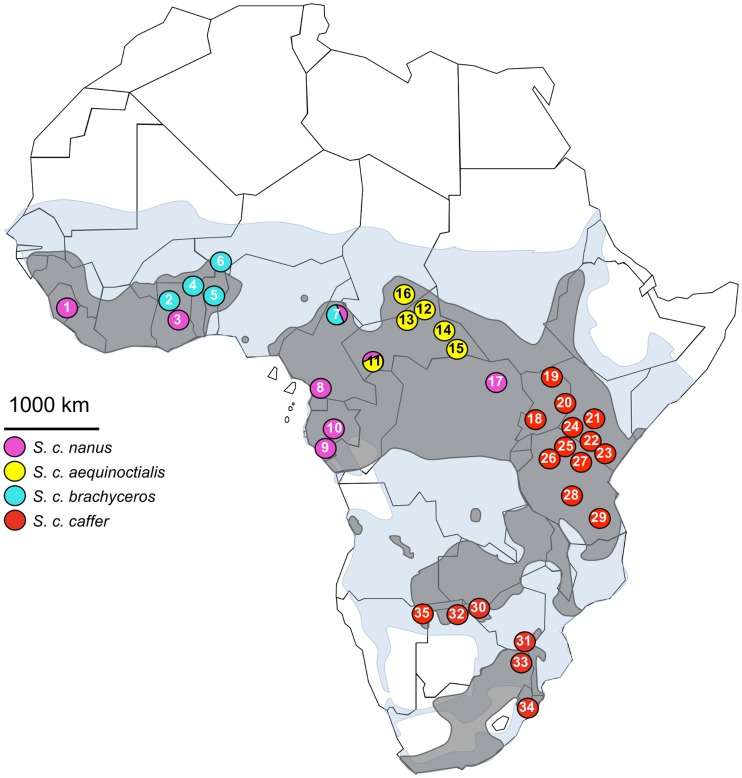
Map of the African continent with sampling origin. Past distribution of the African buffalo is represented in blue (after Furstenburg, personal field notes 1970–2008- unpublished), with an overlapping shape of the actual distribution represented in grey (after the distribution map of the IUCN’s Antelope Specialist Group, 2008). The four subspecies currently recognized based on morphological characteristics were sampled, with the *S. c. nanus* subspecies represented in pink, *S. c. aequinoctialis* in yellow, *S. c. barchyceros* in turquoise, and *S. c. caffer* in red. At locality number 7 and 11, morphological characteristics were intermediate between the *S. c. nanus* and the *S. c. brachyceros*/*S. c. aequinoctialis* subspecies respectively, represented by both color. 1. Gola Forest; 2. Mole; 3. Kpetsu; 4. Arly, Pama, Singou; 5. Pendjari; 6. W; 7. Benoue; 8. Campo ma’an; 9. Gamba; 10. Lope; 11. Ngoto Forest; 12. St-Floris; 13. Bangoran, Koukourou, Sangha; 14. Ouadda, Bria, Ndji River; 15. Mbari; 16. Zakouma; 17. Garamba; 18. Queen Elizabeth, Lake Mburo, Muchison Falls; 19. Kidepo Valley; 20. Mount Elgon; 21. Laikipia; 22. Amboseli, Nairobi; 23. Tsavo; 24. Masai Mara, Nakuru; 25. Serengeti, 26. Maswa; 27. Arusha; 28. Kizigo; 29. Selous; 30. Hwange; 31. Gonarezhou; 32. Chobe; 33. Kruger; 34. Hluhluwe-Imfolozi; 35. Namibia unknown origin.

A set of 787 buffalo samples from West (Ghana, Burkina Faso, Benin, Niger, Sierra Leone, Chad), Central (Cameroon, Central African Republic, Democratic Republic of Congo, Gabon), East (Uganda, Kenya, Tanzania) and Southern Africa (Zimbabwe, Botswana, South Africa, Namibia, Angola) was analyzed. Details and location of the samples are shown in Table 6 and Figure 6. This extensive sampling covers almost the entire distribution of the African buffalo and comprises samples of all putative subspecies. Sample access was facilitated by a large campaign of epidemiological monitoring, but also because buffalo is a prestigious hunting trophy.

Blood, hair, tissue and dung samples were kept in ethanol solution and stored at ambient temperature, except the dung samples from Ghana, Sierra Leone and Benin, which were collected on Whatman FTA cards [80], [81]. Total DNA was extracted using either a DNeasy tissue kit (QIAGEN) or a Puregene kit (QIAGEN), following the manufacturer’s instructions. The Puregene protocol was modified slightly for use with Whatman FTA cards. DNA was released from the cards by soaking 1.25 cm^2^ of the card in 240 µl water and heating this to 90°C for 10 minutes. Following this, 120 µl of the solution was transferred to a new tube together with 480 µl Puregene Cell Lysis solution and 3 µl of a 20 mg/ml proteinase K solution. The remainder of the protocol followed the manufacturer’s instructions.

The *D-loop* hypervariable region (HVR) and adjacent regions were amplified in the majority of samples using forward L0 (5′- CCCAAAGCTGAAATTCTACTTAAACTA-3′) and reverse S0 (5′-TCTAGGCATTTTCAGTGCCTTGCTTT-3′) primers [82]. In the samples from Ghana, Sierra Leone, Benin, Cameroon and Gabon, DNA amplification took place with forward primer Dsca1 (5′-AATATAAAGAGCCTCCCCAG-3′) and reverse primer Dsca2 (5′-CGGCCATAGCTGAGTCC-3′) [35]. In general, DNA amplification was carried out as described in Heller [43] with polymerase chain reaction (PCR) products sent to MACROGEN Inc (Capillary electrophoresis on a 3730XL) or sequenced following Heller [43] protocols. PCR reactions on DNA extracted from dung were performed with a Qiagen Hotstart PCR kit following the manufacturer’s instructions. Negative controls were invariably included to check for contamination. PCR products of DNA extracted from dung were cleaned with a MultiScreen PCR_96_ Cleanup kit (Millipore) and sequenced using a DETT sequencing kit according to the manufacturer’s specifications (GE Healthcare). The resulting sequence PCR products were cleaned with a Montage SEQ_96_ Sequencing Reaction Cleanup kit (Millipore).

Newly sequenced individuals are registered on the National Institutes of Health (NIH) genetic sequence database (GenBank- JQ065982–JQ066169, JQ780485–JQ780603). Previously sequenced data are available on the same sequence database (AF313151–AF313345, JQ403422–JQ403515 and AF028843–AF029038, with exclusion of AF028863, AF028871, AF028876–AF028882, and AF028891 sequences). Seven sequences of *Bubalus bubalis*, the Asian water buffalo, the closest relative to *Syncerus caffer*, were used as an outgroup for our study (Genbank accession numbers: AF475260, AF475278, AY702618, AF547270, EF396999, EF536326, EF536327).

### Sequence Analysis

The resulting sequences were read and aligned using CLUSTAL_X [83], as implemented in BIOEDIT v.7.09 [84] and using Chromas software [85], with corrections by eye. Newly sequenced samples led to the identification of a 604-bp overlapping region. When aligning those newly sequenced HVR region with those obtained from GenBank, a 286-bp overlapping region (including indels) was identified. Nineteen samples were excluded from the final analyses as these contained large amounts of missing data (samples from Mole (n = 1), Niger (n = 5) and CAR (n = 1)), or required the introduction of a large (85 bp) indel in the alignment, corresponding to the conserved region *ETAS2* (Extended Termination Associated Sequence) [86] (Lope NP (n = 10), Kidepo Valley (n = 1) and Angola (n = 1)). In total, 768 samples were included in the final analyses. Because of complications during analyses (see *e.g.* [87] for a discussion of the effect of missing data on analyses), all sites containing alignment gaps or ambiguous nucleotides (*i.e.,* missing data) were removed, which resulted in a final dataset of 195 unambiguous characters. The dataset comprised 116 mutations of which 109 were parsimony informative, with a large number of the variability associated to rare variants that differed in just one nucleotide site each. The transition/transversion ratio was estimated at 9.55∶1.

A haplotype network was constructed using the minimum spanning network method (MINSPNET in ARLEQUIN v.3.1) [88] with default settings. This produces a network representing the most parsimonious relationship between haplotypes. Networks are preferable to trees for intraspecific studies because they do not force haplotypes to occupy tip positions and allow for multifurcations in the topology [89].

#### 1. Population differentiation

The hierarchical distribution of genetic variance among and within populations was assessed using an analysis of molecular variance (AMOVA) on the basis of individual nucleotide frequencies. We also performed pairwise comparisons of individual nucleotide frequencies between populations using Wright’s F-statistics, as implemented in ARLEQUIN v.3.1 [90]. The groups and populations for AMOVA and Wright’s F-statistics were defined according to their geographic position, including the national park or game reserve where it was collected (see Table 6), except for the neighboring populations in the Chobe-Hwange and the Masai Mara-Serengeti-Maswa ecosystems that were not significantly differentiated from one another. The statistical significance of the *F*-statistics was assessed using 1 000 random permutations. For all populations where the number of included samples exceeded four, we calculated the number of haplotypes, haplotype diversity *h* and nucleotide diversity *π* [91] (including standard deviations) using ARLEQUIN and DNaSP [92].

#### 2. Demographic trends

Past demographic history of each of the buffalo lineages was inferred by a pairwise mismatch distribution analysis between individuals [93], comparing the distribution of observed pairwise nucleotide differences, with the expected distribution in an exponentially expanding population. To assess the statistical significance of the distribution, we examined the sum of square deviations (SSD) between the observed and expected mismatch and Harpending’s raggedness index *r* computed under a population growth-decline model in ARLEQUIN. The *P*-value of the test was approximated based on the fraction of times the real data showed a lower value than the simulated data. The timing of demographic expansion can also be roughly estimated by the mode of mismatch distribution *τ* expressed as *τ = *2 *µt*, where *t* is the expansion time in number of generations and *μ* is the mutation rate for the whole sequence [94]. Generation time was fixed within a range of five to seven years, based on the estimates of O’Ryan [34] and Prins (personal communication), and the mutation rate per site was fixed to 32% per million years for the *D-loop* as estimated by Shapiro [95]. Demographic history was also inferred by testing departure from neutrality using Fu’s *Fs* and Tajima D statistics [96] in DNaSP.

#### 3. Coalescence based analyses

BEAST v1.6.1 [97] was used to reconstruct the colonization history of buffalo populations. Recent developments in the software allow one to simultaneously infer the genealogy and ancestral geographic state at each node in the tree [98]. Furthermore, the method allows identification of statistically significant diffusion rates between geographic states (*i.e.,* historical migration patterns) through a proper Bayes factor test rather than by parsimony analysis [98]. We chose to define the country of origin as the geographical states contained within the software as we were interested in spatial patterns at a larger scale (*i.e.,* above the level of the sampling location) (Table 6). This yielded 17 distinct states. We imposed normalized inverse-distance (straight-line distance) based priors on the diffusion rate indicators between countries (*i.e.,* geographical midpoint between sampled regions in each country) to incorporate geographical separation of the samples in the analyses. A strict molecular clock was applied with a normally distributed substitution rate prior, with 95% of the probability density between 2.3×10^−7^ and 4.1×10^−7^, as estimated by Shapiro [96] for the steppe bison *Bison priscus*. Analyses were conducted assuming a constant population size in order to avoid over-parameterization. The best fitting nucleotide substitution model, according to the Akaike information criterion (AIC) [99], was the HKY (Hasegawa, Kishino and Yano) substitution model [100]; we therefore specified this model in all analyses. BEAST MCMC chains were run for 50 million generations, with sampling of statistics and trees every 5 000 steps. Convergence was verified using TRACER v1.4 [97]. A maximum lineage credibility tree was constructed using TreeAnnotator [97], and the geographical state at each node was visualized in FigTree v1.3.1 (Rambaut 2006–2009). Finally, Bayes factors (BF) [98] were calculated to estimate the diffusion rates between states throughout the trees. We imposed a threshold value of BF = 3 to determine the significance of the diffusion process connecting the location states.

An isolation-with-migration (IM) model for two closely related populations or ecophenotypes was applied using the IM program [101], [102]. The IM model presents seven demographic parameters, scaled by the mutation rate: current effective population sizes, ancient effective population size, migration rate in both directions, time of population splitting and a splitting parameter. The latter parameter, not included in newer versions of IM (IMA and IMA2), estimates what fraction of the ancestral population formed each of the two current populations or ecophenotypes. We used a burn-in of 500 000 steps followed by a run of 12–15 million steps. Prior distributions were chosen that included all or most of the range over which the posterior density is non-trivial. We ran the program for a sufficient length of time so that there were no obvious trends in the trend line plots and ensured that the lowest effective sample size (ESS) for estimates exceeded 50. The latter was not always achievable, especially for the time of population splitting, irrespective of the length of the runs. However, we got similar estimates for all parameters in all simulations, providing confidence in our findings. The estimates of the mean and the limits of the 95% confidence interval deviated no more than 27% and 41%, respectively, from their average value across three simulations. We used a geometric heating scheme with *h1* = 0.8 and *h2* = 0.9, applying metropolis coupling using a 10 chain geometric heat mode with 45 chain swap attempts per step. The final prior values used for respectively current population size, ancestral population size, migration rate, lower limit divergence time, and upper limit divergence time were *q_current_* = 70, *q_ancestral_* = 8, *m* = 0.12, *w = *7 and t = 40, respectively. The splitting parameter *s* was included, migration rates were estimated in both directions and the HKY substitution model was applied.

Due to computational constraints, 360 samples were randomly selected, 180 from West-Central and 180 from South-Eastern Africa. Three simulations with 360 samples were performed, each using a different random subset of samples and a different random seed number. All populations within a specific group were represented with a random selection of samples, while equalizing sample size per population as much as possible resulting in the following scheme: West Africa (90 samples), Cameroon and Gabon (54 samples), Chad, Central African Republic and Democratic Republic of Congo (Eastern part of Central Africa close to the lineage border, 36 samples), Uganda (Western part of East Africa close to the lineage border, 36 samples), Kenya and Tanzania (54 samples) and Southern Africa (90 samples). Countries close to the border between two lineages were defined as distinct groups to get maximum sample representation from the presumed center of the divergence.
